# Zolpidem-triggered atrial fibrillation in a patient with cardiomyopathy: a case report

**DOI:** 10.1186/s12872-024-04016-5

**Published:** 2024-07-04

**Authors:** Xiaolin Li, Yunpeng Jin

**Affiliations:** 1 Department of Nutrition, The Fourth Affiliated Hospital of School of Medicine, International School of Medicine, International Institutes of Medicine, Zhejiang University, Yiwu, 322000 China; 2 Department of Cardiology, The Fourth Affiliated Hospital of School of Medicine, International School of Medicine , International Institutes of Medicine, Zhejiang University, Yiwu, 322000 China

**Keywords:** Case report, Zolpidem, Atrial fibrillation, Cardiomyopathy, Duchenne muscular dystrophy

## Abstract

**Background:**

Zolpidem is a non-benzodiazepine hypnotic widely used to manage insomnia. Zolpidem-triggered atrial fibrillation (AF) in patients with cardiomyopathy has never been reported before.

**Case presentation:**

A 40-year-old man with Duchenne muscular dystrophy-related cardiomyopathy attempted suicide and developed new-onset AF after zolpidem overdose. One year before admission, the patient visited our clinic due to chest discomfort and fatigue after daily walks for 1 month; both electrocardiography (ECG) and 24-hour Holter ECG results did not detect AF. After administration of cardiac medication (digoxin 0.125 mg/day, spironolactone 40 mg/day, furosemide 20 mg/day, bisoprolol 5 mg/day, sacubitril/valsartan 12/13 mg/day), he felt better. AF had never been observed before this admission via continuous monitoring during follow-up. Sixteen days before admission, the patient saw a sleep specialist and started zolpidem tartrate tablets (10 mg/day) due to insomnia for 6 months; ECG results revealed no significant change. The night before admission, the patient attempted suicide by overdosing on 40 mg of zolpidem after an argument, which resulted in severe lethargy. Upon admission, his ECG revealed new-onset AF, necessitating immediate cessation of zolpidem. Nine hours into admission, AF spontaneously terminated into normal sinus rhythm. Results from the ECG on the following days and the 24-hour Holter ECG at 1-month follow-up showed that AF was not detected.

**Conclusions:**

This study provides valuable clinical evidence indicating that zolpidem overdose may induce AF in patients with cardiomyopathy. It serves as a critical warning for clinicians when prescribing zolpidem, particularly for patients with existing heart conditions. Further large-scale studies are needed to validate this finding and to explore the mechanisms between zolpidem and AF.

**Supplementary Information:**

The online version contains supplementary material available at 10.1186/s12872-024-04016-5.

## Background

Zolpidem, a non-benzodiazepine hypnotic, is widely used in the pharmacological therapy of insomnia in adults [1]. It has adverse events, including falls and subsequent fractures [2, 3], neuropsychological adverse effects such as parasomnias, amnesia, hallucinations, and suicidality [4, 5], as well as abuse, dependence, and withdrawal [6].

Arrhythmia is also a side effect of zolpidem, although rarely reported. There have been instances of zolpidem causing QT prolongation and provoking ventricular fibrillation [7, 8]. However, atrial fibrillation (AF) as a side effect of zolpidem use remains unreported. Herein, we present a case of a 40-year-old man with Duchenne muscular dystrophy (DMD)-related cardiomyopathy who attempted suicide and developed new-onset AF after zolpidem overdose.

## Case presentation

A 40-year-old man who attempted suicide by overdosing on sleeping pills (zolpidem tartrate tablets, 40 mg) resulting in lethargy for 8 h was admitted to our hospital. Upon presentation, his vital signs were as follows: blood pressure, 91/66 mmHg; irregular heart rate, 54 beats/min; and normal oxygen saturation. The timeline of the case is shown in Table 1.

**Table 1:** The timeline of the case

Date	Episode	Main symptoms	Main examination		Medication
2019-10-07	First	Chest discomfort and fatigue after daily walks for 1 month.	ECG and 24-hour Holter ECG revealed complete right bundle branch block, premature ventricular contractions. AF had never been observed via continuous monitoring during follow-up. Echocardiography revealed diffuse severe hypokinesis with an estimated ejection fraction of 22.7%, enlargement of the left atrium (58.0 mm) and left ventricle (72.7 mm), and mild mitral regurgitation.		Start digoxin (0.125 mg/day), spironolactone (40 mg/day), furosemide (20 mg/day), bisoprolol (5 mg/day), sacubitril/valsartan (12/13 mg/day).
2020-09-18	Second	Insomnia for 6 months.	ECG revealed complete right bundle branch block, premature ventricular contractions.		Start zolpidem tartrate tablets (10 mg/day).
2020-10-04	Third	Attempted suicide by overdosing on sleeping pills (zolpidem tartrate tablets, 40 mg) resulting in lethargy for 8 h.	On arrival	ECG revealed new-onset AF with a heart rate of 54 beats/min. Echocardiography revealed diffuse severe hypokinesis with an estimated ejection fraction of 35%, enlargement of the left atrium (60.2 mm) and left ventricle (73.0 mm), and mild mitral regurgitation.	Stop zolpidem tartrate tablets.
			Nine hours later	ECG revealed complete right bundle branch block, premature ventricular contractions.	He was insisted on medication and regular follow-up after discharge.
		No palpitations and insomnia.	Till date	ECG and 24-hour Holter ECG revealed complete right bundle branch block, premature ventricular contractions. No AF was not detected.	No drug dose adjusted. He became cautious toward sleeping pills.

ECG: electrocardiogram; AF: atrial fibrillation

The patient has suffered from mild skeletal muscle weakness since the age of 15 years and was diagnosed with DMD through dystrophy gene analysis and clinical manifestations. In the patient’s family, four men spanning three generations were also diagnosed with DMD. Additional image files show this in more detail [see Additional file 1]. Multiplex PCR analysis of the dystrophin gene from peripheral blood revealed a deletion of exons 45–49, which applies to all family members (except the patient’s uncle [I 1] for death). Additional image files show this in more detail [see Additional file 2]. There is no involvement of family in this study. There was no other special discovery in the patient’s past history, personal history and family history in addition to the above.

**Fig. 1:**
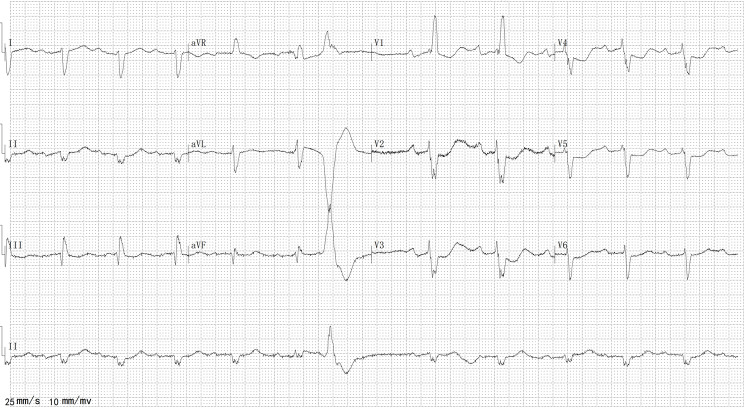
ECG 1 year before this admission

The patient visited our clinic due to chest discomfort and fatigue after daily walks for 1 month since the previous year. His electrocardiography (ECG) (Figs. 1) and 24-hour Holter ECG results revealed a complete right bundle branch block and premature ventricular contractions. Moreover, his ECG results revealed diffuse severe hypokinesis with an estimated ejection fraction of 22.7%, enlarged left atrium (58.0 mm) and left ventricle (72.7 mm), as well as mild mitral regurgitation. The patient regularly took oral medication including digoxin (0.125 mg/day), spironolactone (40 mg/day), furosemide (20 mg/day), bisoprolol (5 mg/day), sacubitril/valsartan (12/13 mg/day). His symptoms improved after taking these medications. AF had never been observed before this admission via continuous monitoring during follow-up.

Sixteen days before this hospital admission, the patient visited a sleep clinic and started zolpidem tartrate tablets (10 mg/day) due to insomnia for 6 months. His ECG results revealed no significant change.

**Fig. 2:**
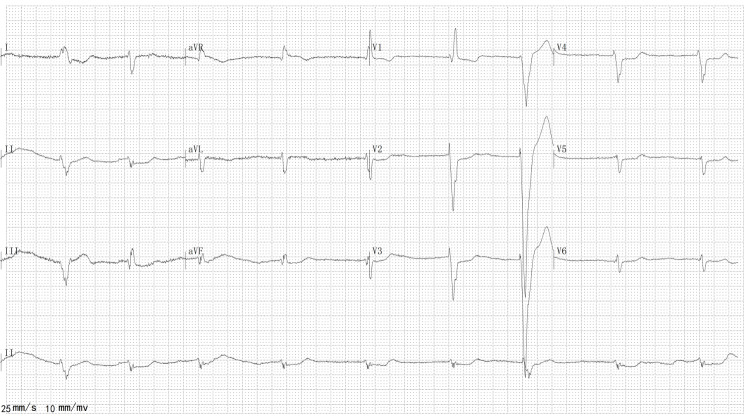
ECG at admission

The night before admission, the patient attempted suicide by taking 40 mg zolpidem tartrate tablets after an argument, resulting in severe lethargy. At admission, his ECG revealed new-onset AF with a heart rate of 54 beats/min (Fig. 2) and diffuse severe hypokinesis with an estimated ejection fraction of 35%, enlargement of the left atrium (60.2 mm) and left ventricle (73.0 mm), as well as mild mitral regurgitation, roughly the same as before. Notable laboratory test values showed no significant dynamic change before and after admission: an elevated creatine kinase, 1059 U/L; creatine kinase-MB, 41.7 U/L; troponin-I, 0.82 ng/ml; and N-terminal pro B-type natriuretic peptide, 911.66 ng/L. Zolpidem was stopped immediately, heart rhythm was monitored by telemetry, and i.v. fluids were administered upon admission. The patient stabilized 9 h after admission, and AF spontaneously terminated into normal sinus rhythm. The medication history was carefully confirmed by the patient and his family. His body mass index (BMI) was 23.9 kg/m^2^. Cognitive behavioral therapy was performed to improve his insomnia, and he was discharged 3 days after admission. According to the ECG monitor during the next few days and the 24-hour Holter ECG at 1-month follow-up, his heart rate maintained a sinus rhythm, and no AF was detected.

Causality, severity, and preventability assessment of the adverse drug reaction (ADR) are shown in Table 2. According to the Naranjo causality scale and World Health Organization Uppsala Monitoring Center (WHO-UMC) assessment scale, this ADR and zolpidem may be associated. The Hartwig’s severity scale score indicated a moderate severity level for the reaction (level 4b). The ADR may indeed have been preventable based on the modified Schoumock and Thornton scale [9].

**Table 2:** Causality, severity, and preventability assessment of the adverse drug reaction

Parameter	Observation
Naranjo causality scale	Probable (7)
WHO-UMC assessment scale	Probable
Hartwig’s severity scale	Moderate, level 4b
Severity of the reaction	Hospitalization
Outcome	Recovered
Predisposing factors	Overdose
Modified Schoumock and Thornton scale	Preventable

WHO-UMC: World Health Organization Uppsala Monitoring Center

All appropriate patient consent forms for images and other clinical information to be reported in the journal were obtained for this case report. The patients understand that their names and initials will not be published and due efforts will be made to conceal their identity, but anonymity cannot be guaranteed.

## Discussion and conclusions

Patients with DMD experience evolving insomnia throughout their lifetimes [10]. It is common to identify DMD patients experiencing chronic insomnia who also suffer from other psychiatric comorbidities such as mood and anxiety disorders [11]. Certain medications can be used to augment nonpharmacologic interventions to manage insomnia. Non-benzodiazepine hypnotics, including zolpidem, are commonly prescribed by physicians [12]. Clinically, zolpidem is widely used in patients with insomnia, including DMD patients. However, DMD patients have a high risk of suicide due to the possible coexistence of other psychiatric disorders, as in our patient. Nonpharmacologic treatment strategies are preferred for this condition.

Zolpidem is associated with arrhythmia. Two instances have indicated that zolpidem could contribute to a prolonged QTc and ventricular fibrillation. However, AF after zolpidem use has not been reported before.

Over the past hundred years, AF is the arrhythmia that has been studied the most among all other heart rhythm disorders [13]. However, crucial questions regarding the formation and perpetuation of the disease remain unanswered. To the best of our knowledge, atrial fibrosis, inflammation, oxidative stress, aging, arterial hypertension, obesity, excessive alcohol use, diabetes mellitus, certain medications, obstructive sleep apnea (OSA), and genetic factors contribute to the development of AF [14]. Other causes such as electrolyte disorders, intracranial pressure elevation, hyperthyroidism, infection, and hyperthermia have been ruled out in the present case. Results of the causality analysis of the new-onset AF are shown in Table 3.

**Table 3:** Causality analysis of the new-onset AF

Causes	Observation	Possibility
Medication	Zolpidem overdose	Probable
	Digoxin	Doubtful
	Spironolactone	Doubtful
	Furosemide	Doubtful
	Bisoprolol	Doubtful
	Sacubitril/valsartan	Doubtful
Atrial fibrosis, inflammation, and oxidative stress	Cardiomyopathy: considered stable	Possible
Aging	40 years old	Doubtful
Arterial hypertension	Denied	Doubtful
Obesity	Not observed	Doubtful
Excessive alcohol use	Denied	Doubtful
Diabetes mellitus	Denied	Doubtful
Obstructive sleep apnea	Not observed	Doubtful
Genetic factors	No family history of AF	Doubtful

AF: atrial fibrillation

In this case, the underlying disease (DMD-related cardiomyopathy) and combined medication may increase the risk of AF.

In the current scenario, cardiomyopathy was considered stable based on the consistent clinical manifestations and ECG and laboratory test results at admission. New-onset heart failure or any other types of arrhythmia had not been observed. AF had not been detected before. After stopping zolpidem, AF subsequently terminated spontaneously into normal sinus rhythm and did not recur. Therefore, the association between cardiomyopathy and new-onset AF remains unclear.

The patient attempted suicide after a family argument, indicating that psychological stress might have contributed to the occurrence of atrial fibrillation. However, the conclusion about the correlation between psychological factors and AF remains controversial [15, 16]. The current European Society of Cardiology guideline on AF mentions psychological distress and emotional fluctuations as known consequences of AF only but not as risk factors [17].

The patient has been taking normal doses of digoxin, spironolactone, furosemide, bisoprolol, and sacubitril/valsartan since the previous year before admission. The combined effect of these drugs on heart rhythm could be complex. Digoxin, spironolactone, furosemide, bisoprolol, and sacubitril/valsartan are commonly considered therapeutic drugs for patients with heart failure with reduced ejection fraction, but not as risk factors for inducing AF [18]. Zolpidem has few drug interactions. The patient was taking multiple cardiac medications, and so far, no interactions between these drugs and zolpidem have been observed [19]. Existing evidence suggests that the coadministration of digoxin does not alter the pharmacokinetics of zolpidem [20]. Zolpidem was primarily suspected due to its overdose proximate to the new-onset AF; once withdrawn, the patient’s AF resolved. Therefore, the overdose on zolpidem corresponds as the most probable cause of AF based on the Naranjo causality scale and WHO-UMC assessment scale.

Zolpidem could have caused AF via respiratory depression. DMD and insomnia are both commonly comorbid with OSA [21, 22]. We are not sure if the patient has OSA, but certainly the high dose of zolpidem could have caused it because of pharyngeal muscle relaxation and delayed arousal worsening hypoxaemia [23]. Obstructed inspiration generates large negative intrathoracic pressure fluctuations, leads to acute atrial distension, shortens atrial refractoriness, slows conduction, and increases the occurrence of intraatrial conduction block in humans. These acute transient arrhythmogenic changes during apnea may contribute to the development of AF [24]. In addition, zolpidem can decrease central respiratory drive, increase upper airway resistance, decrease inspiratory flow, decrease carbon dioxide ventilatory response, and increase blood carbon dioxide concentration [25]. Hypercapnia increases effective refractory period in action potentials and conduction in the atria which can increase vulnerability to AF in pre-existing atrial myopathy [26], which was very likely present in this case with atrial dilation.

Several observational epidemiological studies have produced conflicting results on the relationship between hypnotic usage and heart disease. A meta-analysis of observational epidemiological studies supported that zolpidem showed a decreased risk (− 29%) of developing or dying from heart disease, but benzodiazepines were associated with an increased risk (80%) of or mortality from heart disease [27]. Exposure to benzodiazepines was associated with increased cardiovascular mortality (HR: 1.65; 95% CI 1.39, 1.97) in a large population study with 85,353 women, but not when further adjusted for antidepressant use (HR: 1.15; 95% CI 0.94, 1.40), nor in the multivariable model (HR: 0.93; 95% CI 0.75, 1.16) [28]. Therefore, it is still unknown whether zolpidem could increase the risk of AF.

In this case, it is of interest that only 40 mg zolpidem induced severe lethargy and new-onset AF, and this dose is generally not considered toxic. On the contrary, a study on the subjective effects of zolpidem showed that 20 mg was considered “pleasant” and gave a “high” despite zolpidem increased ratings of “sleepy” [29]. When given in a nightly dosage of up to 20 mg, zolpidem is generally well tolerated by patients with insomnia, only 5.2% of them resulted in lethargy [30]. Once the patient stabilized after admission, the patient and his family denied intake of other toxic substances. Because zolpidem is metabolized through cytochrome P450, while bisoprolol [31], digoxin [32], spironolactone [33], and furosemide [34] have the same metabolic pathway through cytochrome P450, co-application of these medications may result in impaired metabolism of affected compounds and elevate zolpidem plasma concentrations toward critical levels. The presence of cardiomyopathy that reduce the repolarization reserve is expected to precipitate AF during zolpidem treatment.

Another thing worth mentioning is that the new-onset AF was accompanied by a slow ventricular rate. This patient had right bundle branch block and probably conductive system disorder (not uncommon in patients with DMD), which we believe was the main reason for the slow heart rate during AF. Moreover, a rat study supported the fluctuation of heart rate would be attenuated with a higher dose of zolpidem [35]. As in this case, the slow ventricular rate of AF may also associated with the zolpidem overdose.

The main limitation of this case report is the lack of toxicity analyses and recurrence of ADR with rechallenge, which would not be ethical. Patients with any form of cardiomyopathy have a high risk of AF. We cannot completely rule out accidental detection of AF during admission in this case.

This case suggests that zolpidem overdose could increase the risk of AF in patients with cardiomyopathy, as indicated by both causality analysis and explainable mechanism. It serves as a critical warning for clinicians when prescribing zolpidem, particularly for patients with existing heart conditions. Further large-scale studies are needed to validate this finding and to explore the mechanisms between zolpidem and AF.

### Electronic supplementary material

Below is the link to the electronic supplementary material.

Supplementary Material 1

Supplementary Material 2

Supplementary Material 3

## Data Availability

The data underlying this article will be shared on reasonable request with the corresponding author.

## Abbreviations

AF: Atrial fibrillation
ECG: Electrocardiogram
DMD: Duchenne muscular dystrophy
ADR: Adverse drug reaction
WHO-UMC: World Health Organization Uppsala Monitoring Center
OSA: Obstructive sleep apnea
