# Supplementary material for: Zolpidem-triggered atrial fibrillation in a patient with cardiomyopathy: a case report
Source: BMC Cardiovasc Disord. 2024 Jul 4;24:339. doi: 10.1186/s12872-024-04016-5 (PMC11225507; doi:10.1186/s12872-024-04016-5)
Supplement: Supplementary file 1 — Supplementary Material 1 [file 12872_2024_4016_MOESM1_ESM.docx]

**Additional files**

Additional file 1.tif: Pedigree of the family with Duchenne muscular dystrophy genes. I = first generation, II = second generation, III = third generation, arrow = proband.

Additional file 2.tif: Multiplex PCR analysis of dystrophin gene from peripheral blood of the patient.
