# Supplementary figures and images for: Zolpidem-triggered atrial fibrillation in a patient with cardiomyopathy: a case report
Source: BMC Cardiovasc Disord. 2024 Jul 4;24:339. doi: 10.1186/s12872-024-04016-5 (PMC11225507; doi:10.1186/s12872-024-04016-5)

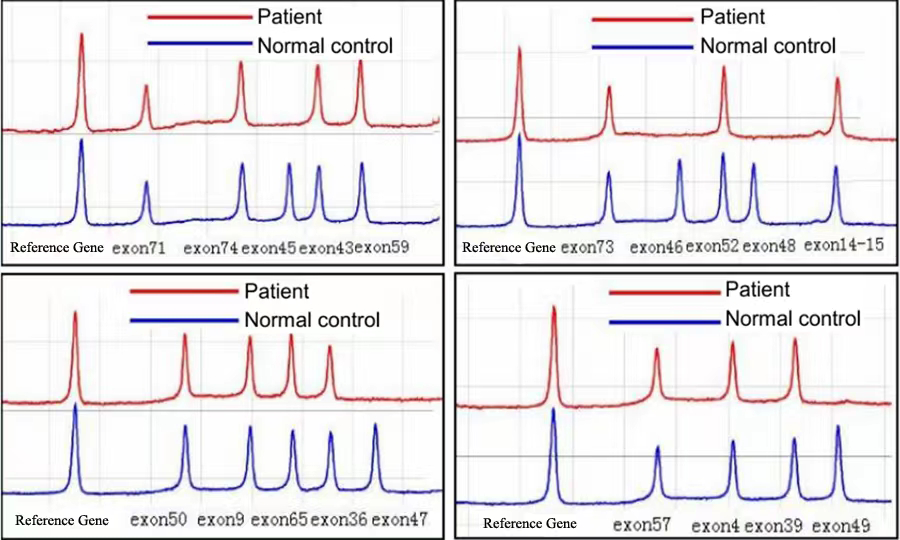

Supplement: Supplementary file 2 — Supplementary Material 2 [file 12872_2024_4016_MOESM2_ESM.tif]

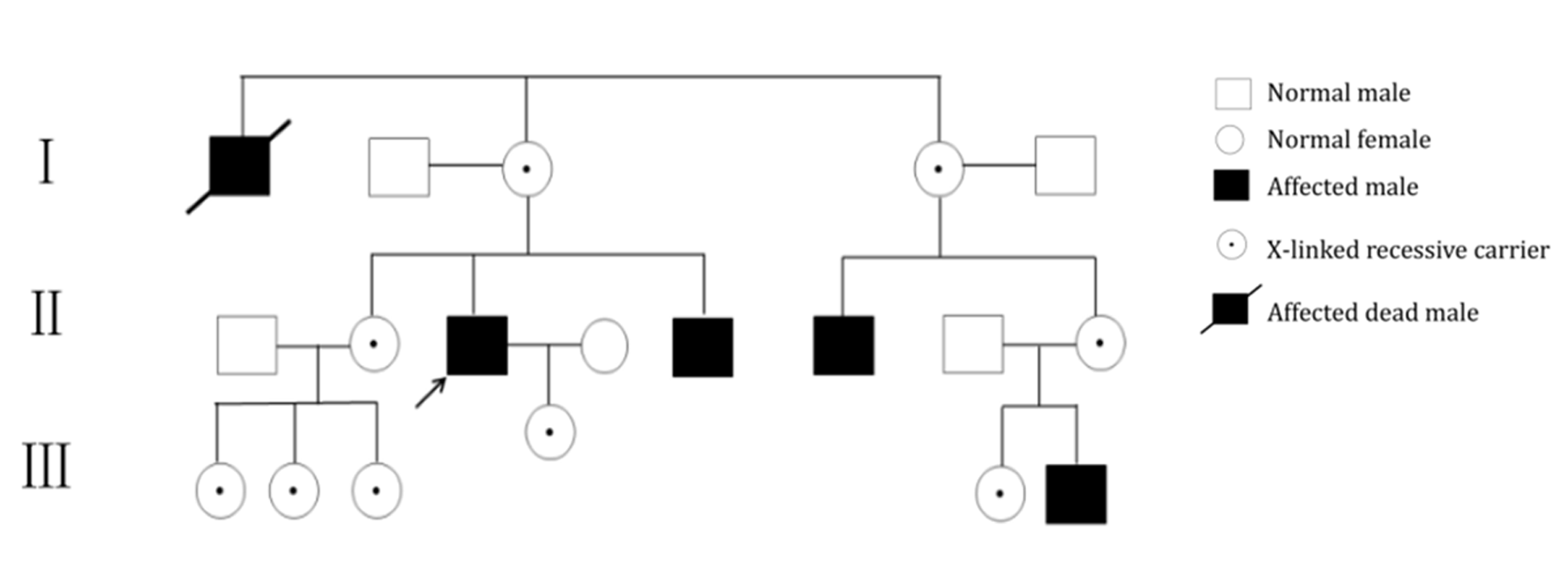

Supplement: Supplementary file 3 — Supplementary Material 3 [file 12872_2024_4016_MOESM3_ESM.tif]
